# Supplementary material for: Knowledge gaps and positive attitudes toward adult vaccination among nursing students: A Cross-sectional study
Source: PLoS One. 2026 May 26;21(5):e0350295. doi: 10.1371/journal.pone.0350295 (PMC13210314; doi:10.1371/journal.pone.0350295)
Supplement: S1 File — (DOCX) [file pone.0350295.s002.docx]

**S1 File. Questionnaire**

*As part of the continuous improvement and implementation of the internal quality assurance system, it is proposed to analyse the knowledge, attitude and main sources of information available to Nursing students.*

*Of course, the questionnaire is confidential, in accordance with the provisions of Organic Law 3/2018, of 5 December, on Personal Data Protection and guarantee of digital rights. The study will be carried out according to the principles of the Declaration of Helsinki on human clinical trials (Ref No. 579/06/2020) and was approved by the Ethics Committee of the Cardenal-Herrera University (CEEI23/426).*

*Your questionnaire will be mixed with those of many other students, so no one will ever know what your answers were.*

*Apart from being confidential, your collaboration in the survey is completely voluntary.*

*The approximate estimated time to complete the questionnaire is 6 minutes.*

*Thank you in advance for your participation.*

*Please start the survey.*

☐ **By ticking this box you consent to participation in the study. Furthermore, you declare that you are informed of your rights of access and information, rectification, deletion, oblivion, limitation of processing, data portability and objection, which you may exercise by writing to: jesus.sanchez@uchceu.es**

**Section A**

*Sociodemographic characteristics*

**1**. **Academic year. Indicate the higher course in which you are enrolled.**

☐ First

☐ Second

☐ Third

☐ Fourth

**2. Sex:**

☐ Female

☐ Male

**3. Age:**____years

**4. Marital status:**

☐ Single

☐ Married or cohabiting couple

**5 Do you have healthcare experience?**

☐ Yes

☐ No

**6. If you have experience in the healthcare sector, how many years of experience do you have?**

_______years

**Section B**

*Knowledge of the vaccination schedule and training received on vaccines*

**1. Is there a vaccination schedule developed by the spanish Ministry of Health for the adult population (≥15 years)?**

☐ Yes

☐ No

☐ Do not know

**2. If yes, are you aware of the vaccines included in the vaccination schedule for adults?**

☐ Yes

☐ No

**3. How would you rate the training you have received so far in your degree programme on vaccines?**

☐ Nothing at all

☐ Some

☐ Neither much nor little

☐ Quite a lot

☐ A lot

**4. Do you believe that the general public receives sufficient information about vaccines before they are administered?**

☐ Yes

☐ No

☐ Do not know

**Section C**

*General opinion on vaccines and their recommendation.*

**1. Overall, your opinion on vaccines as a whole is:?**

☐ Not favourable

☐ Indifferent

☐ Favourable

☐ Very Favourable

**2. Do you consider vaccines to be safe?**

☐ Nothing at all

☐ Something

☐ Neither much nor little

☐ Quite a lot

☐ A lot

**3. Do you consider vaccines to be effective in preventing diseases?**

☐ Nothing at all

☐ Something

☐ Neither much nor little

☐ Quite a lot

☐ A lot

**4. In your opinion, the vaccines in the vaccination schedule should be**

☐ Compulsory

☐ Voluntary

**5. As a future healthcare professional, would you recommend vaccination to your patients?**

☐ No

☐ Yes, to a specific vaccine

☐ Yes, to all vaccines

**6. Have you encountered resistance from other professionals when recommending vaccines?**

☐ Yes

☐ No

☐ Do not know

**7. If you have encountered resistance from other professionals, could you indicate what reason they gave or suggested for not recommending vaccination? (mark as many answers as you wish)**

☐ I have not encountered resistance from other professionals.

☐ He/She was not concerned about the spread of the disease and its natural progression.

☐ He/She had doubts about the safety of the vaccine.

☐ He/She had doubts about the efficacy of the vaccine.

☐ The patient did not belong to any risk group.

☐ He/She had doubts about the composition of the vaccine.

☐ He/She had doubts about vaccines in general.

☐ Other reasons

**8.** **What is your usual source for information on vaccines? (mark as many answers as you wish)**

☐ Spanish Agency for Medicines and Health Products (AEMS), technical sheet

☐ Media (tv, press, magazines, etc)

☐ Internet search (Websites, forums, blogs)

☐ Social networks (YouTube, Twitter, Facebook, etc)

☐ Scientifics bases (Pubmed, Web of Science, Scopus, etc)

☐ I ask my doctor or nurse

☐ I ask colleagues/tutor/teaching collaborator

☐ Institutional websites (Ministry of Health, Autonomous Regions, International Agencies, etc)

**9**. **What is the source you think the general population turns to for information on vaccines?** **(mark as many answers as you wish)**

☐ Spanish Agency for Medicines and Health Products (AEMS), technical sheet

☐ Media (tv, press, magazines, etc)

☐ Internet search (Websites, forums, blogs)

☐ Social networks (YouTube, Twitter, Facebook, etc)

☐ Scientifics bases (Pubmed, Web of Science, Scopus, etc)

☐ They ask their doctor or nurse

☐ They ask colleagues/tutor/teaching collaborator

☐ Institutional websites (Ministry of Health, Autonomous Regions, International Agencies, etc)

**THANK YOU FOR YOUR COLLABORATION**
