# Supplementary material for: Knowledge gaps and positive attitudes toward adult vaccination among nursing students: A Cross-sectional study
Source: PLoS One. 2026 May 26;21(5):e0350295. doi: 10.1371/journal.pone.0350295 (PMC13210314; doi:10.1371/journal.pone.0350295)
Supplement: S2 File — (DOCX) [file pone.0350295.s001.docx]

**S2 File. Data related to Figures 1 and 2.**

To ensure full transparency and facilitate exact replication of the results, the complete response counts and percentages from the survey questions underlying Figures 1 and 2 are presented in the tables below

Figure 1. Nursing students' self-reported sources of information on vaccinations and their perceptions of sources used by the general population.

| **Student reference resources** | **%** | **n** |
| --- | --- | --- |
| I do not consult information about vaccines. | 8,1 | 23 |
| Institutional websites | 27,8 | 156 |
| I ask colleagues/tutor/teaching collaborator | 29,7 | 167 |
| I ask my doctor or nurse | 54,1 | 304 |
| Social networks | 10,7 | 60 |
| Scientifics bases | 18,1 | 102 |
| Internet search | 37,7 | 212 |
| Media | 21,2 | 119 |
| AEMS, technical sheet | 23.0 | 129 |

| **Sources of reference for the general population as perceived by nursing students** | **%** | **n** |
| --- | --- | --- |
| They do not consult information about vaccines. | 4,6 | 13 |
| Institutional websites | 7,3 | 41 |
| They ask colleagues/tutor/teaching collaborator | 15,5 | 87 |
| They ask their doctor or nurse | 39,3 | 221 |
| Scientifics bases | 3,9 | 22 |
| Social networks | 48,4 | 272 |
| Internet search | 62,3 | 350 |
| Media | 48,8 | 274 |
| AEMS, technical sheet | 8,4 | 47 |

Institutional websites: Ministry of Health, Autonomous Regions, International Agencies; Scientific bases: Pubmed, Web ofScience, Scopus, etc.; Social networks: YouTube, Twitter, Facebook, etc.; Internet search: websites, forums, blogs; Media: tv, press, magazines, etc.; AEMS: Spanish Agency for Medicines and Health Products.

**Figure 2.** Perception of reasons for resistance to vaccine recommendations by other health professionals detected by nursing students. Data shown as a percentage of positive responses and calculated based on 269 students who reported encountering resistance from other professionals when recommending vaccines.

| **Reasons for resistance** | **%** | **n** |
| --- | --- | --- |
| Other | 13,9 | 22 |
| He/She had doubts about vaccines in general. | 15,6 | 68 |
| He/She had doubts about the composition of the vaccine. | 16,3 | 71 |
| The patient did not belong to any risk group. | 5,7 | 25 |
| He/She had doubts about the efficacy of the vaccine. | 29,9 | 130 |
| He/She had doubts about the safety of the vaccine. | 40,9 | 178 |
| He/She was not concerned about the spread of the disease and its natural progression. | 8,0 | 35 |
| I have not encountered any resistance. | 41,5 | 233 |
