# Supplementary material for: Knowledge gaps and positive attitudes toward adult vaccination among nursing students: A Cross-sectional study
Source: PLoS One. 2026 May 26;21(5):e0350295. doi: 10.1371/journal.pone.0350295 (PMC13210314; doi:10.1371/journal.pone.0350295)
Supplement: Attachment — Submitted filename: Research report.pdf [file pone.0350295.s003.pdf]

Research report

Research title

**Nursing Students' Knowledge Shortcomings and Supportive Attitudes on Adult Immunization: Insights from a Cross-Sectional Descriptive Investigation in Alicante,
Spain**

First: Positives of the Research

1- Importance of the Topic

- The importance of this topic lies in the fact that adult vaccination is essential for prevention and reducing complications, yet it remains below the required level due to low awareness and hesitancy. Studying nursing students is particularly important because they are future leaders who will be influential in raising awareness and promoting vaccines, thus supporting improved training and curricula. However, focusing solely on this group without addressing broader factors such as policies, vaccine availability, and media influence may limit the effectiveness of the approach, potentially hindering its generalization.

2- Diversity of Data Collection Tools

- The advantages of using a variety of data collection tools lie in its ability to triangulate results and reduce bias associated with relying on a single tool. Combining standardized questionnaires, interviews/focus groups, and observation or record reviews—where available—allows for a more comprehensive measurement of knowledge, attitudes, and actual behavior, while also improving the validity of results and enhancing their contextual interpretation. However, an excessive number of tools without standardizing concepts, testing the validity and reliability of each tool, or without a clear data integration plan can produce inconsistent data, weaken comparability, and increase the burden on participants. Therefore, the choice of each tool should be justified and directly linked to the research questions and the analytical methodology.

3- Integrating Theory with Practice

- Integrating theory with practice is crucial because it translates concepts (such as models for changing behavior and vaccine hesitancy) into practical applications like patient communication techniques and misinformation correction, thereby strengthening the interpretation of results and making recommendations actionable. However, this integration can be weakened if theory remains general without being translated into measurable variables and clear, evaluable training steps.

4- Educational and Affective Impact

- The educational and affective impact is that it enhances knowledge, improves attitudes, confidence, and professional readiness to communicate with patients

and recommend vaccines, and reveals training gaps for curriculum development. However, it may be influenced by self-assessment and social bias and does not demonstrate actual behavioral change without pre-/post-assessment or follow-up.

5- Clarity of Analysis

- The clarity of the analysis is achieved when the data processing steps and the selection of analytical methods are clearly presented and justified, and the results are presented in an organized manner linked to the research questions, thus enhancing confidence and reproducibility. However, ambiguity in defining variables, handling missing data, or presenting results without linking them to hypotheses weakens the strength of the analysis.

6 - Discussion of the results

- The discussion of the results shows that nursing students' attitudes toward vaccines and their recommendation of them appear generally positive. However, this coincides with significant knowledge gaps—particularly regarding adult vaccination—suggesting a clear need to strengthen vaccinology content within curricula and clinical training early and systematically. The discussion also connects these findings to previous literature to illustrate their consistency with a broader pattern of discrepancies between positive attitudes and knowledge levels and suggests practical implications for the quality of health education and addressing vaccine hesitancy. Nevertheless, it remains necessary to refine generalizations and more specifically attribute any differences between groups to contextual factors (such as varying educational exposure and information sources).

Second: Points to improve research quality

1- Title and Abstract:

- The title should accurately reflect the study's main variables (knowledge, attitudes, and adult vaccination), target group, and methodological design, while avoiding generality or unnecessary length. The abstract should be well-organized and clear, including a brief background, the study's objective and design, sample size, measurement tools, key quantitative findings with their statistical significance, and practical conclusions without overgeneralization. It is also preferable to specify the study's timeframe and location in the abstract to enhance transparency and comprehensibility from the first reading.

2- Introduction and Research Gap:

- The introduction should be logically structured, beginning with a brief overview of the importance of adult vaccination and its associated challenges. It should then move to the role of nursing students as future healthcare professionals, drawing on recent and directly relevant literature. It is also advisable to identify the research gap more explicitly, clarifying what previous studies have not addressed (such as a lack of local data, limited focus on adult vaccination specifically, or a lack of connection between knowledge and attitudes). Clearly highlighting this gap and linking it to the study's objectives strengthens the

scientific rationale and enhances the coherence of the research's theoretical framework.

3. Theoretical Framework and Hypothesis Development:

- The theoretical framework should be strengthened by adopting a clear explanatory model (such as models of health behavior or vaccine hesitancy) that guides the selection of variables and measurement tools, while also clarifying how theoretical concepts are translated into measurable indicators within the study. It is also advisable to formulate specific and direct hypotheses that express expected relationships between knowledge, attitudes, and demographic or educational variables, rather than relying solely on general descriptive objectives. Developing theoretically grounded hypotheses enhances methodological coherence and strengthens the explanatory power of the results.

4. Data Collection and Measurement Tools:

- A more detailed description of the data collection tools is recommended, including the source of the questionnaire or measurement instrument, any cultural adaptation measures, and validity and reliability checks (such as internal consistency coefficients). The sampling method, inclusion and exclusion criteria, and the distribution and collection methods should also be clearly explained to minimize the potential for bias. Enhanced transparency in these areas increases the reliability and reproducibility of the results and improves the quality of measurement and statistical inference.

7. Validity, Reliability, and Discrimination:

- The presentation of evidence for the validity and reliability of the instrument should be strengthened, such as statements of content and construct validity, and the provision of internal consistency coefficients (e.g., Cronbach's alpha) or test-retest results where possible. It is also advisable to demonstrate the instrument's ability to distinguish between different levels of knowledge or attitudes (discrimination validity), and to indicate whether the items can differentiate between groups with varying characteristics (e.g., academic years). Systematically presenting these indicators enhances the credibility of the results and strengthens the inferences drawn from the data.

8. Statistical Analysis:

- It is advisable to provide a more detailed explanation of the statistical analysis plan, justifying the choice of tests based on the nature and level of measurement of the variables, and verifying their underlying assumptions. The method for handling missing data should also be indicated, and measures of effect size and confidence intervals should be presented alongside statistical significance values to enhance the scientific interpretation of the results. If multiple analyses are conducted, it is preferable to clearly explain the procedures for controlling Type I errors to ensure the accuracy and reproducibility of the conclusions.

9. Discussion and Contributions:

- The discussion should clearly highlight the study's scientific contribution by linking the findings to the theoretical framework and previous literature and demonstrating their theoretical or practical applications (at the level of nursing education or vaccination policies). It is also advisable to clarify the practical value of the findings in developing curricula or health education strategies, distinguishing between local relevance and generalizability. Furthermore, the methodological limitations should be presented objectively, and future research directions directly based on the findings should be proposed, thus strengthening the study's academic contribution.

10. Ethical considerations:

- Ethical considerations should be clearly outlined, including obtaining approval from a research ethics committee, explaining the mechanism for obtaining informed consent from participants, guaranteeing confidentiality and anonymity, and outlining data storage and protection mechanisms. It is also advisable to clarify that participation is voluntary and that participants have the right to withdraw without repercussions, and to disclose any potential conflicts of interest or funding sources. Presenting these elements transparently enhances the credibility of the study and ensures adherence to established ethical standards in health research.

11. Presentation, Language, and Formatting:

- It is advisable to improve the overall presentation by organizing subheadings consistently and ensuring accurate numbering of tables, figures, and references within the text. The language should also be reviewed for academic accuracy to avoid repetition, vague or imprecise phrasing, and to ensure consistency in the use of scientific terminology. Strict adherence to the journal's guidelines regarding documentation style, the presentation of statistics, and word count in different sections is recommended to enhance the research's professionalism and publishability without stylistic flaws.

12. References

- It is advisable to update the bibliography to include recent studies directly relevant to adult vaccination and the knowledge and attitudes of nursing students, while avoiding excessive reliance on outdated or generic sources. It is also essential to ensure strict adherence to the journal's documentation style, with all in-text references matching the bibliography without omission or duplication. Furthermore, it is recommended to cite both international and local studies to balance a global perspective with a regional context, thereby strengthening the theoretical framework and enhancing the research's credibility.

Final Evaluation: Acceptance with Major Revisions

Reason:

- After reviewing the research, I believe it addresses a topic of clear scientific and applied importance and possesses a sound methodological foundation.

However, several key aspects require development before its acceptance for publication. These include strengthening the theoretical framework and formulating the hypotheses more clearly, providing a more detailed presentation of the measurement tools and their psychometric properties, clarifying the rationale for the statistical analysis, and delving deeper into the discussion of the results and their connection to the literature in a more critical manner. Therefore, the study does not suffer from any definitive methodological shortcomings that would warrant rejection, but it requires substantial revisions to enhance its scientific quality and contribution. I thus recommend its acceptance conditional upon major revisions.

Thank you very much
