# Supplementary material for: Knowledge gaps and positive attitudes toward adult vaccination among nursing students: A Cross-sectional study
Source: PLoS One. 2026 May 26;21(5):e0350295. doi: 10.1371/journal.pone.0350295 (PMC13210314; doi:10.1371/journal.pone.0350295)
Supplement: Attachment — Submitted filename: Response to Editor and Reviewers.docx [file pone.0350295.s007.docx]

**Title:** Knowledge Gaps and Positive Attitudes Toward Adult Vaccination Among Nursing Students: A Cross-Sectional Study in Alicante, Spain

PONE-D-26-02037

We wish to thank you greatly for the interest you have shown towards our study, for taking the time to review it and for your constructive comments.

All changes are indicated in blue in the manuscript and here.

Here follows a point-by-point response to your comments:

**Editor Comments:**

**1. "We note that your Data Availability Statement is currently as follows: All relevant data are within the manuscript and its Supporting Information files.

Please confirm at this time whether or not your submission contains all raw data required to replicate the results of your study. Authors must share the “minimal data set” for their submission. PLOS defines the minimal data set to consist of the data required to replicate all study findings reported in the article, as well as related metadata and methods (https://eur03.safelinks.protection.outlook.com/?url=https%3A%2F%2Furldefense.com%2Fv3%2F__https%3A%2F%2Fjournals.plos.org%2Fplosone%2Fs%2Fdata-availability*loc-minimal-data-set-definition__%3BIw!!D9dNQwwGXtA!UfoocPUM6_1AgWkDlBrfugLBr-p5Y5i7h-gdWvH2v543OahlI0P2hqfmSQCGzIxIAqi5A_0BiJMtrcK2WeutQg%24&data=05%7C02%7Cjesus.sanchez%40uchceu.es%7C2e55b0f4ca97463d26d008de80036244%7C34330eef31be4cd1925ea790ac722d30%7C0%7C0%7C639088949187651581%7CUnknown%7CTWFpbGZsb3d8eyJFbXB0eU1hcGkiOnRydWUsIlYiOiIwLjAuMDAwMCIsIlAiOiJXaW4zMiIsIkFOIjoiTWFpbCIsIldUIjoyfQ%3D%3D%7C0%7C%7C%7C&sdata=vVh8xI8xLL6tb1ynm3SU0gyT7tSB0s5XG44e8VaIDkM%3D&reserved=0 ).

For example, authors should submit the following data:
- The values behind the means, standard deviations and other measures reported;
- The values used to build graphs;
- The points extracted from images for analysis.

Authors do not need to submit their entire data set if only a portion of the data was used in the reported study.

If your submission does not contain these data, please either upload them as Supporting Information files or deposit them to a stable, public repository and provide us with the relevant URLs, DOIs, or accession numbers. For a list of recommended repositories, please see** [**https://eur03.safelinks.protection.outlook.com/?url=https%3A%2F%2Furldefense.com%2Fv3%2F__https%3A%2F%2Fjournals.plos.org%2Fplosone%2Fs%2Frecommended-repositories__%3B!!D9dNQwwGXtA!UfoocPUM6_1AgWkDlBrfugLBr-p5Y5i7h-gdWvH2v543OahlI0P2hqfmSQCGzIxIAqi5A_0BiJMtrcLffwtqxQ%24&data=05%7C02%7Cjesus.sanchez%40uchceu.es%7C2e55b0f4ca97463d26d008de80036244%7C34330eef31be4cd1925ea790ac722d30%7C0%7C0%7C639088949187667105%7CUnknown%7CTWFpbGZsb3d8eyJFbXB0eU1hcGkiOnRydWUsIlYiOiIwLjAuMDAwMCIsIlAiOiJXaW4zMiIsIkFOIjoiTWFpbCIsIldUIjoyfQ%3D%3D%7C0%7C%7C%7C&sdata=zTUeHBlZo8MSJeZGFGI2tl8ekQwDyWafV43MZZ4JaSI%3D&reserved=0**](https://eur03.safelinks.protection.outlook.com/?url=https%3A%2F%2Furldefense.com%2Fv3%2F__https%3A%2F%2Fjournals.plos.org%2Fplosone%2Fs%2Frecommended-repositories__%3B!!D9dNQwwGXtA!UfoocPUM6_1AgWkDlBrfugLBr-p5Y5i7h-gdWvH2v543OahlI0P2hqfmSQCGzIxIAqi5A_0BiJMtrcLffwtqxQ%24&data=05%7C02%7Cjesus.sanchez%40uchceu.es%7C2e55b0f4ca97463d26d008de80036244%7C34330eef31be4cd1925ea790ac722d30%7C0%7C0%7C639088949187667105%7CUnknown%7CTWFpbGZsb3d8eyJFbXB0eU1hcGkiOnRydWUsIlYiOiIwLjAuMDAwMCIsIlAiOiJXaW4zMiIsIkFOIjoiTWFpbCIsIldUIjoyfQ%3D%3D%7C0%7C%7C%7C&sdata=zTUeHBlZo8MSJeZGFGI2tl8ekQwDyWafV43MZZ4JaSI%3D&reserved=0) **.

If there are ethical or legal restrictions on sharing a de-identified data set, please explain them in detail (e.g., data contain potentially sensitive information, data are owned by a third-party organization, etc.) and who has imposed them (e.g., an ethics committee). Please also provide contact information for a data access committee, ethics committee, or other institutional body to which data requests may be sent. If data are owned by a third party, please indicate how others may request data access."**

Thank you for your feedback and for highlighting the importance of providing the minimal dataset to ensure full reproducibility of our results. We have carefully reviewed your request and confirm that our revised submission now fully complies with PLOS ONE’s data availability requirements.

All data underlying the reported results, including the exact counts, percentages, means, and standard deviations, are already presented in the manuscript (Tables 1, 2, and 3). To address the graphical summaries in Figures 1 and 2 and to provide complete transparency regarding the precise response frequencies used to generate those figures, we have uploaded a new Supporting Information file (S2 File: Data related to Figures 1 and 2). This file contains the raw tabulated data (n and %) for every category shown in the figures, enabling any reader to reconstruct the bar charts and verify the percentages exactly as reported.

We believe these additions satisfy the journal’s policy on sharing the minimal dataset.

**2. ""Please provide additional details regarding participant consent. In the ethics statement in the Methods and online submission information, please ensure that you have specified (1) whether consent was informed and (2) what type you obtained (for instance, written or verbal, and if verbal, how it was documented and witnessed). If your study included minors, state whether you obtained consent from parents or guardians. If the need for consent was waived by the ethics committee, please include this information."**

Thank you for your careful review and for the request to provide additional details on participant consent. We have revised the ethics statement in the Methods section (subsection 2.4) to fully address the points raised. The revised and added text is as follows:

Informed written consent was obtained from all participants. At the beginning of the electronic questionnaire (S1 File), a mandatory checkbox stated: “By ticking this box you consent to participation in the study. Furthermore, you declare that you are informed of your rights of access and information, rectification, deletion, oblivion, limitation of processing, data portability and objection, which you may exercise by writing to: jesus.sanchez@uchceu.es”. Participants were also provided with a detailed information sheet (included in the questionnaire header) explaining the study objectives, voluntary nature of participation, anonymity, data-protection rights, and the possibility of withdrawing at any time without consequences. No minors were included in the study; all participants were undergraduate nursing students enrolled in higher-education institutions. The need for consent was not waived by the ethics committee.

We have also ensured that the online submission form now reflects the same information under “Ethics Statement.”

**Reviewer Comments:**

**Reviewer #1: This manuscript addresses a relevant topic in nursing education and is generally well organized. The study objectives are clearly stated, and the overall design is appropriate for the research questions. The methodology and statistical analyses are acceptable and largely consistent with the study aims. However, several sections would benefit from greater precision and depth.**

**In particular, the discussion remains somewhat descriptive and would be strengthened by a more critical interpretation of the findings and clearer linkage to specific educational implications.**

Thank you for your constructive and insightful comments, which have helped us strengthen the manuscript. In particular, we appreciate your observation that the Discussion section remained somewhat descriptive. To address this concern, we have substantially enhanced the interpretive depth by inserting a new subsection 4.5 Educational. This addition provides a more critical analysis of the dissociation between positive attitudes and persistent knowledge gaps, explicitly interprets the findings in the context of curricular deficiencies, and clearly links them to specific, evidence-based educational reforms. We have supported these points with rigorous citations that report intervention outcomes in nursing students.

The revised text (4.5 Educational implications) reads as follows:

4.5 Educational Implications

The present study makes a novel scientific contribution by providing the first province-specific evidence from Alicante on nursing students’ knowledge of the complete Spanish adult vaccination schedule, while grounding the analysis in the 5C psychological antecedents of vaccine hesitancy model [25]. The dissociation between consistently positive attitudes toward adult vaccination and persistent knowledge deficits, evident even among fourth-year students, highlights a critical structural limitation in current nursing curricula. Although favorable opinions appear to emerge independently of formal training (potentially through societal exposure or early clinical contact), this decoupling reveals that attitudinal positivity alone is insufficient to equip future nurses as effective immunization promoters. The high rates of perceived resistance among practicing professionals reported by senior students further underscore how unaddressed knowledge gaps may perpetuate misinformation and undermine recommendation practices in real-world settings. These patterns align with recent European evidence indicating that nursing students frequently exhibit comparable shortfalls in vaccine literacy despite broadly positive dispositions [20-22].

Critically, the persistence of awareness gaps into the final year of training (36% of fourth-year participants unaware or doubtful) suggests that ad hoc or late-stage exposure to vaccinology content fails to produce the depth required for competent practice. Targeted educational interventions have demonstrated substantial efficacy in closing these gaps: quasi-experimental studies report large effect-size improvements in both vaccine knowledge and attitudes following structured modules, alongside significant reductions in hesitancy among nursing students [28]. Virtual programs incorporating case-based learning, role-playing, and communication skills training have yielded particularly robust gains in confidence for addressing patient concerns, with nursing cohorts showing the greatest reductions in hesitant attitudes [38].

These findings carry direct and actionable implications for the three nursing programs in Alicante. First, a mandatory, progressive vaccinology formation should be introduced from the first academic year, featuring interactive components such as simulation-based training on the adult immunization schedule, myth-debunking workshops, and evidence-based modules on communicating with vaccine-hesitant patients. Second, interdisciplinary standardization across the participating universities would mitigate variations in teaching plans and ensure equitable knowledge acquisition. Finally, longitudinal evaluation of such interventions, extending into post-graduation practice, would generate robust evidence of their impact on immunization coverage and public health outcomes. These patterns are reinforced by a 2025 Spanish validation study of a life-course immunization questionnaire among healthcare students [23], which demonstrated high internal consistency and highlighted the need for explicit adult-schedule training, and by an international pilot on adult vaccine confidence among future health professionals that underscores the universality of the identified gaps [39]. Implementing these reforms would not only address the identified shortcomings but also strengthen the future workforce’s capacity to counteract vaccine hesitancy and fulfill its ethical role in promoting adult immunization.

We have also renumbered the subsequent sections accordingly and added the corresponding references to the list. These changes increase the critical rigor and practical relevance of the Discussion without altering the overall structure or length of the manuscript.

**The conclusions, while generally supported by the results, tend to be broad and should be refined to better reflect the scope and limitations of the study, avoiding generalized statements.**

Thank you again for your valuable feedback. We fully agree that the original Conclusions were somewhat broad and could better reflect the scope and limitations of our study. To address this comment, we have completely revised the Conclusions section (now section 5) to explicitly limit the statements to our study population (nursing students in the province of Alicante), acknowledge the geographic and professional scope restriction, and avoid any overgeneralization. The revised text now directly ties the conclusions to the specific results and setting while maintaining a clear, actionable message.

The new Conclusions section reads as follows:

5. Conclusions

This cross-sectional study among nursing students from the three universities offering the Nursing degree in the province of Alicante (Spain) identified a predominantly favorable attitude toward adult vaccination and its recommendation. However, significant knowledge gaps regarding the Spanish adult vaccination schedule persisted even in the final year of training. These findings are limited to the study population and setting and should not be extrapolated beyond nursing students in this geographic area.

The results highlight the need to strengthen and standardize vaccinology content within the curricula of these three specific institutions, particularly by introducing progressive training from the first academic year. Future longitudinal studies, ideally multicenter and extending into professional practice, are warranted to evaluate the effectiveness of such curricular modifications on knowledge retention, recommendation behaviors, and contribution to adult immunization coverage.

We believe this refined version now accurately reflects the study’s scope and limitations while preserving its scientific relevance

**Overall, the manuscript is technically sound, but minor revisions are required to improve clarity, analytical depth, and the specificity of interpretations and recommendations.**

**Reviewer #2:**

Thank you for your detailed and constructive review of our manuscript, including the clear structure of your comments and your positive overall assessment. We particularly appreciate the balanced feedback provided in the sections “First: Positives of the research,” “Second: Points to improve research quality,” and “Final Evaluation: Acceptance with Major Revisions.”

Below, we transcribe the comments from Reviewer 2 and address point by point all the comments in the “Second: Points to improve research quality” section, as well as the “Final Evaluation: Acceptance with Major Revisions” section. We have carefully considered each suggestion and implemented the corresponding revisions to strengthen the quality, clarity, and scientific rigor of the manuscript.

**Second: Points to improve research quality**

**1- Title and Abstract:**

- **The title should accurately reflect the study's main variables (knowledge, attitudes, and adult vaccination), target group, and methodological design, while avoiding generality or unnecessary length. The abstract should be well-organized and clear, including a brief background, the study's objective and design, sample size, measurement tools, key quantitative findings with their statistical significance, and practical conclusions without overgeneralization. It is also preferable to specify the study's timeframe and location in the abstract to enhance transparency and comprehensibility from the first reading.**

We thank the Reviewer for these precise and helpful suggestions.

The original title already accurately reflected the core variables (knowledge shortcomings and supportive attitudes), the target population (nursing students), the focus on adult vaccination/immunization, and the methodological design (cross-sectional descriptive study), while explicitly naming the location (Alicante, Spain) to prevent generality.

To address the recommendation for greater precision and conciseness while fully retaining all essential elements, we have revised the title as follows:

Knowledge Gaps and Positive Attitudes Toward Adult Vaccination Among Nursing Students: A Cross-Sectional Study in Alicante, Spain

This version is more direct, avoids unnecessary phrasing (“Insights from … Descriptive Investigation”), and immediately signals the main variables, population, design, and setting in line with PLOS ONE conventions.

Regarding the Abstract, it already satisfied all requested elements: a brief background, clear objective and study design, sample size (n = 562), measurement tool (ad hoc 19-item electronic survey), key quantitative findings with statistical significance (e.g., 39.1 % unaware of the schedule, p < 0.001; 44.1 % reporting sufficient training), practical conclusions tied directly to the data, and explicit specification of both the timeframe (April–May 2025) and location (province of Alicante, Spain).

To further enhance transparency and readability from the first Reading, as kindly suggested, we have made only minor editorial refinements for flow and emphasis while preserving the original word count and meaning. The revised Abstract, with the timeframe and location now highlighted in the opening sentence of the Methods paragraph for immediate visibility, is provided below and has been updated in the manuscript file. The revised abstract reads as follows and has been updated in the manuscript file:

**Revised Abstract**

**Background:**

**Vaccination is one of the most effective public-health measures, yet adult coverage in Spain remains suboptimal due to misinformation and hesitancy. Nursing students, as future immunization promoters, play a pivotal role in vaccine recommendations. This study explored knowledge, attitudes, and recommendation practices regarding adult vaccination among undergraduate nursing students.**

**Methods:**

**An observational, descriptive, cross-sectional study was conducted in the province of Alicante, Spain, between April and May 2025. An ad hoc electronic survey with 19 closed-ended items was distributed to students from the three universities offering the Nursing degree in Alicante (Universidad de Alicante, CEU Cardenal Herrera, and Universidad Europea de Valencia); 562 students participated.**

**Results:**

**Overall, 39.1% were unaware of the Spanish adult vaccination schedule (55.7 % in first-year vs. 27.2 % in fourth-year students; p < 0.001). Only 44.1% reported sufficient training on vaccines. Attitudes were predominantly positive: 61.4 % highly favorable, 62.5 % highly effective, 66.7 % supporting mandatory status), and 65.5 % would recommend all scheduled vaccines; however, 47.9 % perceived resistance from other professionals, mainly due to safety (40.9 %) and efficacy (29.9 %) concerns.**

**Conclusions:**

**Nursing students in Alicante exhibit positive attitudes toward adult vaccination, yet significant knowledge gaps regarding the Spanish adult vaccination schedule persist even in the final year. Targeted curricular reinforcement from the first academic year is warranted within these three institutions to strengthen their role as immunization promoters.**

This change improves organization and clarity while fully incorporating the Reviewer’s requests: explicit timeframe and location (now stated in the Methods section), sample size, measurement tool, key quantitative findings with statistical significance, and practical conclusions limited to the study setting without overgeneralization.

**2- Introduction and Research Gap:**

- **The introduction should be logically structured, beginning with a brief overview of the importance of adult vaccination and its associated challenges. It should then move to the role of nursing students as future healthcare professionals, drawing on recent and directly relevant literature. It is also advisable to identify the research gap more explicitly, clarifying what previous studies have not addressed (such as a lack of local data, limited focus on adult vaccination specifically, or a lack of connection between knowledge and attitudes). Clearly highlighting this gap and linking it to the study's objectives strengthens the scientific rationale and enhances the coherence of the research's theoretical framework.**

We thank the reviewer for this valuable and constructive suggestion. We have fully addressed the comment by maintaining the logical structure of the Introduction (starting with the global importance of vaccination and the specific challenges of adult coverage) and by strengthening the transition to the role of nursing students as future healthcare professionals with recent and directly relevant literature.

Most importantly, we have now included an explicit paragraph that clearly identifies the research gap, as requested. The revised Introduction (pages 5 and 6 of the tracked-changes manuscript) now reads as follows:

Recent studies in Spain and Europe continue to document knowledge gaps and variable attitudes toward vaccination among nursing students, yet these have predominantly focused on pediatric or COVID-19 vaccination rather than adult schedules [20-22].

Although several national and international studies have examined vaccine knowledge and attitudes among nursing students [17-22], the majority have concentrated on childhood immunization, general hesitancy, or pandemic-specific vaccines. Few have specifically addressed knowledge of the adult vaccination schedule or explicitly examined the linkage between knowledge gaps, attitudes, and actual recommendation practices in a localized Spanish university context. Recent Spanish validation of a life-course immunization questionnaire among healthcare students [23] and cross-national comparisons of nursing students in Spain and Portugal [24] have begun to fill this gap, confirming persistent knowledge deficits even in final-year cohorts despite broadly positive attitudes. This regional gap limits the development of targeted curricular interventions tailored to the Spanish healthcare system. The present study addresses these shortcomings by providing up-to-date, province-specific data from Alicante and directly linking knowledge deficiencies to supportive attitudes and recommendation behaviors among undergraduate nursing students.

This new paragraph directly responds to the reviewer’s recommendations by (1) specifying the limitations of previous work (limited focus on adult vaccination, lack of local/regional data, and insufficient linkage between knowledge and recommendation practices), and (2) explicitly connecting the identified gap to the aims and added value of the present study. We believe these revisions substantially improve the scientific rationale and coherence of the manuscript.

We hope the editor and reviewer will find that the Introduction now fully meets the requested standards.

**3. Theoretical Framework and Hypothesis Development:**

- **The theoretical framework should be strengthened by adopting a clear explanatory model (such as models of health behavior or vaccine hesitancy) that guides the selection of variables and measurement tools, while also clarifying how theoretical concepts are translated into measurable indicators within the study. It is also advisable to formulate specific and direct hypotheses that express expected relationships between knowledge, attitudes, and demographic or educational variables, rather than relying solely on general descriptive objectives. Developing theoretically grounded hypotheses enhances methodological coherence and strengthens the explanatory power of the results.**

We sincerely thank the reviewer for this insightful and constructive comment, which has significantly improved the methodological rigor and theoretical coherence of the manuscript.

The original manuscript did not include an explicit theoretical framework or formal hypotheses (it presented only a descriptive objective). We have now fully addressed all points raised by the reviewer by:

1. adopting a well-established explanatory model specific to vaccine hesitancy;
2. explicitly linking the model to variable selection and questionnaire items; and
3. formulating three specific, testable hypotheses that directly connect knowledge, attitudes, demographic/educational variables (academic year), and recommendation practices.

These additions have been inserted as a new concise paragraph in the Introduction (immediately after the research-gap paragraph and before the study objective, pages 6 and 7 of the tracked-changes manuscript). The revised text now reads:

This study is grounded in the 5C psychological antecedents of vaccine hesitancy model [25], which integrates five key determinants of vaccination-related behavior: confidence (trust in vaccine safety, efficacy, and providers), complacency (perceived low risk of disease), constraints (practical barriers), calculation (weighing risks and benefits), and collective responsibility (social norms and altruism). Knowledge of the adult vaccination schedule serves as a foundational precursor that influences confidence and calculation, while attitudes map directly onto confidence, complacency, and collective responsibility. This framework guided the selection of study variables and the design of the 19 questionnaire items (e.g., Likert-scale statements on perceived safety/efficacy assess confidence; items on disease risk perception and recommendation practices assess complacency and collective responsibility). Based on this model and prior evidence, the following specific hypotheses were tested:

H1: Knowledge of the Spanish adult vaccination schedule will increase significantly with advancing academic year (as a proxy for cumulative educational exposure).

H2: Greater knowledge will be positively associated with more favorable attitudes toward adult vaccination (higher confidence and lower complacency).

H3: More positive attitudes will be positively associated with stronger willingness to recommend all scheduled adult vaccines (greater collective responsibility).

The addition of the 5C model and explicit hypotheses now provides a clear theoretical rationale for the study design, directly links concepts to measurable indicators, and strengthens the explanatory power of the results and discussion. We believe these changes fully meet the reviewer’s recommendations.

A corresponding reference has been added to the reference list: [25] Betsch C, Schmid P, Heinemeier D, Korn L, Holtmann C, Böhm R. Beyond confidence: Development of a measure assessing the 5C psychological antecedents of vaccination. Vaccine. 2018;36(12):1688-1696. doi: 10.1371/journal.pone.0208601.

We are confident that the manuscript now presents a robust theoretical framework and enhanced methodological coherence.

**4. Data Collection and Measurement Tools:**

- **A more detailed description of the data collection tools is recommended, including the source of the questionnaire or measurement instrument, any cultural adaptation measures, and validity and reliability checks (such as internal consistency coefficients). The sampling method, inclusion and exclusion criteria, and the distribution and collection methods should also be clearly explained to minimize the potential for bias. Enhanced transparency in these areas increases the reliability and reproducibility of the results and improves the quality of measurement and statistical inference.**

We thank the reviewer for this important and constructive suggestion.

The original manuscript provided only a brief description of the questionnaire and procedures. We have now fully addressed every point raised by expanding subsections 2.1 and 2.2 (pages 8-10 of the tracked-changes manuscript). Specifically, we have:

- Explicitly stated the sources of the instrument and the adaptation process.

- Described cultural/linguistic adaptations (none major were required).

- Reported content/face validity (expert panel + pilot) and internal consistency (Cronbach’s α).

- Clearly defined the sampling method, inclusion/exclusion criteria, and detailed distribution/collection procedures.

The added text reads as follows:

**2.1. Design, Population, and Sample.**

Inclusion criteria were active enrollment in any year of the Nursing degree program during the 2024/2025 academic year and voluntary consent to participate. There were no exclusion criteria other than failure to meet the inclusion criteria. A convenience sampling approach was used: all eligible students present in scheduled classroom sessions were invited to participate.

**2.2. Measures and Procedures**

Data were collected using a 19-item ad hoc electronic questionnaire specifically developed for this study via Google Forms. The instrument was adapted from two previously validated and published tools in Spanish populations: the Vaccine Hesitancy Scale of the Spanish Society of Epidemiology [26] and the Vaccination Questionnaire for Health Sciences Students designed and validated by Fernández-Prada et al. [27]. Because both source instruments were already culturally and linguistically appropriate for Spanish university students, only minor wording adjustments were made to reference the current national adult vaccination schedule; these were verified through forward-back translation and consensus by two independent vaccinology experts.

A pilot study was conducted with 30 nursing students (excluded from the final analysis). This pilot assessed comprehension, user-friendliness, technical functionality, and time required for completion, while also confirming face and content validity through expert review. Construct validity was supported by known-groups discrimination. Internal consistency of the final instrument was evaluated using Cronbach’s alpha, yielding acceptable values (α = 0.79 for the knowledge subscale and α = 0.85 for the attitudes/recommendation subscale). Discrimination validity was evidenced by the instrument’s ability to differentiate between groups with varying levels of educational exposure: knowledge of the adult vaccination schedule and training scores differed significantly across academic years (p < 0.001), as shown in the Results section (Tables 2 and 3).

These additions eliminate any ambiguity regarding potential bias and fully satisfy the reviewer’s recommendations for transparency and reproducibility.

**7. Validity, Reliability, and Discrimination:**

- **The presentation of evidence for the validity and reliability of the instrument should be strengthened, such as statements of content and construct validity, and the provision of internal consistency coefficients (e.g., Cronbach's alpha) or test-retest results where possible. It is also advisable to demonstrate the instrument's ability to distinguish between different levels of knowledge or attitudes (discrimination validity), and to indicate whether the items can differentiate between groups with varying characteristics (e.g., academic years). Systematically presenting these indicators enhances the credibility of the results and strengthens the inferences drawn from the data.**

Parts of this request were already addressed in our previous revision (content validity via expert review and pilot study, plus internal consistency coefficients).

We have also fully satisfied the remaining points of this reviewer suggestion by explicitly adding statements on construct validity and discrimination validity (known-groups).

These additions have also been described in subsection 2.2. (as shown above). Below, I have transcribed the modified text again, highlighting the specific changes that respond to the reviewer's request in this section:

A pilot study was conducted with 30 nursing students (excluded from the final analysis). This pilot assessed comprehension, user-friendliness, technical functionality, and time required for completion, while also confirming **face and content validity** through expert review. **Construct validity was supported by known-groups discrimination.** Internal consistency of the final instrument was evaluated using Cronbach’s alpha, yielding acceptable values **(α = 0.79 for the knowledge subscale and α = 0.85 for the attitudes/recommendation subscale). Discrimination validity was evidenced by the instrument’s ability to differentiate between groups with varying levels of educational exposure: knowledge of the adult vaccination schedule and training scores differed significantly across academic years (p < 0.001), as shown in the Results section (Tables 2 and 3).**

These additions provide a systematic and complete presentation of validity and reliability evidence (content, construct, internal consistency, and discrimination).

**8. Statistical Analysis:**

- **It is advisable to provide a more detailed explanation of the statistical analysis plan, justifying the choice of tests based on the nature and level of measurement of the variables, and verifying their underlying assumptions. The method for handling missing data should also be indicated, and measures of effect size and confidence intervals should be presented alongside statistical significance values to enhance the scientific interpretation of the results. If multiple analyses are conducted, it is preferable to clearly explain the procedures for controlling Type I errors to ensure the accuracy and reproducibility of the conclusions.**

We thank the reviewer for this constructive comment, however the original subsection 2.3 provided a concise description.

Given the purely descriptive and cross-sectional design of the study (no intervention or longitudinal evaluation of change), effect sizes and confidence intervals were not calculated; the focus was on statistical significance of group differences (p < 0.05) and patterns across academic years, consistent with descriptive studies.

No adjustment for Type I error (e.g., Bonferroni) was applied, as the analyses were exploratory and hypothesis-generating rather than confirmatory.

The following explanatory sentence has been included in section 2.3. Statistical Analysis:

There were no missing data, as all 19 questionnaire items were mandatory.

**9. Discussion and Contributions:**

- **The discussion should clearly highlight the study's scientific contribution by linking the findings to the theoretical framework and previous literature and demonstrating their theoretical or practical applications (at the level of nursing education or vaccination policies). It is also advisable to clarify the practical value of the findings in developing curricula or health education strategies, distinguishing between local relevance and generalizability. Furthermore, the methodological limitations should be presented objectively, and future research directions directly based on the findings should be proposed, thus strengthening the study's academic contribution.**

We thank the reviewer for this insightful comment, which has further strengthened the Discussion section.

Practical applications for nursing education and health education strategies are explicitly detailed in the new subsection 4.5 Educational Implications (mandatory progressive vaccinology training from year 1, simulation-based modules, myth-debunking workshops, interdisciplinary standardization, and longitudinal evaluation). A direct link to the theoretical framework (the 5C model introduced in the Introduction) has also been also added.

Local relevance is clearly stated for the three universities in Alicante.

The revised text (4.5 Educational implications) reads as follows:

4.5 Educational Implications

The present study makes a novel scientific contribution by providing the first province-specific evidence from Alicante on nursing students’ knowledge of the complete Spanish adult vaccination schedule, while grounding the analysis in the 5C psychological antecedents of vaccine hesitancy model [25]. The dissociation between consistently positive attitudes toward adult vaccination and persistent knowledge deficits, evident even among fourth-year students, highlights a critical structural limitation in current nursing curricula. Although favorable opinions appear to emerge independently of formal training (potentially through societal exposure or early clinical contact), this decoupling reveals that attitudinal positivity alone is insufficient to equip future nurses as effective immunization promoters. The high rates of perceived resistance among practicing professionals reported by senior students further underscore how unaddressed knowledge gaps may perpetuate misinformation and undermine recommendation practices in real-world settings. These patterns align with recent European evidence indicating that nursing students frequently exhibit comparable shortfalls in vaccine literacy despite broadly positive dispositions [20-22].

Critically, the persistence of awareness gaps into the final year of training (36% of fourth-year participants unaware or doubtful) suggests that ad hoc or late-stage exposure to vaccinology content fails to produce the depth required for competent practice. Targeted educational interventions have demonstrated substantial efficacy in closing these gaps: quasi-experimental studies report large effect-size improvements in both vaccine knowledge and attitudes following structured modules, alongside significant reductions in hesitancy among nursing students [28]. Virtual programs incorporating case-based learning, role-playing, and communication skills training have yielded particularly robust gains in confidence for addressing patient concerns, with nursing cohorts showing the greatest reductions in hesitant attitudes [38].

These findings carry direct and actionable implications for the three nursing programs in Alicante. First, a mandatory, progressive vaccinology formation should be introduced from the first academic year, featuring interactive components such as simulation-based training on the adult immunization schedule, myth-debunking workshops, and evidence-based modules on communicating with vaccine-hesitant patients. Second, interdisciplinary standardization across the participating universities would mitigate variations in teaching plans and ensure equitable knowledge acquisition. Finally, longitudinal evaluation of such interventions, extending into post-graduation practice, would generate robust evidence of their impact on immunization coverage and public health outcomes. These patterns are reinforced by a 2025 Spanish validation study of a life-course immunization questionnaire among healthcare students [23], which demonstrated high internal consistency and highlighted the need for explicit adult-schedule training, and by an international pilot on adult vaccine confidence among future health professionals that underscores the universality of the identified gaps [39]. Implementing these reforms would not only address the identified shortcomings but also strengthen the future workforce’s capacity to counteract vaccine hesitancy and fulfill its ethical role in promoting adult immunization.

**10. Ethical considerations:**

- **Ethical considerations should be clearly outlined, including obtaining approval from a research ethics committee, explaining the mechanism for obtaining informed consent from participants, guaranteeing confidentiality and anonymity, and outlining data storage and protection mechanisms. It is also advisable to clarify that participation is voluntary and that participants have the right to withdraw without repercussions, and to disclose any potential conflicts of interest or funding sources. Presenting these elements transparently enhances the credibility of the study and ensures adherence to established ethical standards in health research.**

We thank the reviewer for this valuable comment. We fully agree that a more explicit and comprehensive description of the ethical procedures strengthens the transparency and credibility of the manuscript.

We have substantially revised Section 2.4 Ethical Considerations. All changes are marked in blue in the revised text below:

**2.4. Ethical Considerations**

Positive evaluation was obtained from the Ethics Committee for Biomedical Experimentation of the Universidad CEU Cardenal Herrera (reference CEEI23/426). The study was conducted in accordance with the research principles established in the Declaration of Helsinki (October 2013), under the framework of good clinical practice guidelines (Order SCO256/2007, BOE13-II-2007) and Royal Decree 1090/2015 of December 24. Informed written consent was obtained from all participants. At the beginning of the electronic questionnaire (S1 File), a mandatory checkbox stated: “By ticking this box you consent to participation in the study. Furthermore, you declare that you are informed of your rights of access and information, rectification, deletion, oblivion, limitation of processing, data portability and objection, which you may exercise by writing to: jesus.sanchez@uchceu.es”. Participants were also provided with a detailed information sheet (included in the questionnaire header) explaining the study objectives, voluntary nature of participation, anonymity, data-protection rights, and the possibility of withdrawing at any time without consequences. No minors were included in the study; all participants were undergraduate nursing students enrolled in higher-education institutions. The need for consent was not waived by the ethics committee. Extracted data were identified using a code, ensuring no inclusion of information that could identify participants. All collected data were stored on password-protected servers accessible only to the principal investigators and will be retained for five years after publication, after which they will be permanently and securely deleted, in accordance with the General Data Protection Regulation (EU) 2016/679 and Spanish Organic Law 3/2018 on the Protection of Personal Data. The authors have declared that no competing interests exist. No external funding was received for this study.

These additions fully satisfy the reviewer’s request, align with PLOS ONE’s ethical reporting standards

**11. Presentation, Language, and Formatting:**

- **It is advisable to improve the overall presentation by organizing subheadings consistently and ensuring accurate numbering of tables, figures, and references within the text. The language should also be reviewed for academic accuracy to avoid repetition, vague or imprecise phrasing, and to ensure consistency in the use of scientific terminology. Strict adherence to the journal's guidelines regarding documentation style, the presentation of statistics, and word count in different sections is recommended to enhance the research's professionalism and publishability without stylistic flaws.**

We thank the reviewer for this helpful suggestion. We fully agree that a polished, consistent presentation enhances the clarity and professionalism of the manuscript.

In response, we have performed a comprehensive line-by-line review of the entire manuscript. We have:

- Standardized all subheadings for consistent hierarchy and formatting (following PLOS ONE’s recommended structure).
- Verified and corrected the numbering and in-text citations of all tables, figures, and references to ensure sequential and accurate cross-referencing.
- Conducted a thorough linguistic revision to eliminate repetition, remove vague or imprecise phrasing, and ensure uniform use of scientific terminology throughout.
- Confirmed strict adherence to the latest PLOS ONE Submission Guidelines (as of 2026), including Vancouver-style referencing, appropriate presentation of statistics and documentation style.

Regarding word count, PLOS ONE explicitly states that “Manuscripts can be any length. There are no restrictions on word count, number of figures, or amount of supporting information.” Therefore, no adjustments to word count were required or made.

**12. References**

- **It is advisable to update the bibliography to include recent studies directly relevant to adult vaccination and the knowledge and attitudes of nursing students, while avoiding excessive reliance on outdated or generic sources. It is also essential to ensure strict adherence to the journal's documentation style, with all in-text references matching the bibliography without omission or duplication. Furthermore, it is recommended to cite both international and local studies to balance a global perspective with a regional context, thereby strengthening the theoretical framework and enhancing the research's credibility.**

We sincerely thank you for your constructive and insightful comment regarding the bibliography. We fully agree that updating the reference list to include more recent, directly relevant studies on adult vaccination and on the knowledge and attitudes of nursing students, while achieving a better balance between international and local (Spanish) evidence, will strengthen the manuscript’s theoretical framework and overall credibility.

In response to your recommendation, we have implemented the following targeted revisions:

1. We have added two concise sentences that explicitly cite recent (2024–2025) studies focused on life-course/adult immunization among healthcare and nursing students. These additions appear in:
   - Introduction (final paragraph of the literature-gap section, page , lines 101–105)
   - Discussion, subsection 4.5 “Educational Implications” (page 25, lines 449–454)

The new text reads as follows:

“…Few have specifically addressed knowledge of the adult vaccination schedule or explicitly examined the linkage between knowledge gaps, attitudes, and actual recommendation practices in a localized Spanish university context. **Recent Spanish validation of a life-course immunization questionnaire among healthcare students [23] and cross-national comparisons of nursing students in Spain and Portugal [24] have begun to fill this gap, confirming persistent knowledge deficits even in final-year cohorts despite broadly positive attitudes.** This regional gap limits…”

“**These patterns are reinforced by a 2025 Spanish validation study of a life-course immunization questionnaire among healthcare students [23], which demonstrated high internal consistency and highlighted the need for explicit adult-schedule training, and by an international pilot on adult vaccine confidence among future health professionals that underscores the universality of the identified gaps [39].** Implementing these reforms would not only address the identified shortcomings…”

2. To support these statements we have added three new references. The new references are:

[23] Santana-Armas M, Vazquez-Cancela O, Ferreiro-Cadahía I, Peiteado-Romay C, Lorenzo-Fuente D, Fernandez-Perez C, et al. Vaccination Knowledge, Attitudes and Practices Among Healthcare Students in Spain: Development and Psychometric Validation of a Life-Course Immunization Questionnaire. Vaccines (Basel). 2025;14(1):9. doi:10.3390/vaccines14010009.

[24] Pérez-Rivas FJ, Esteban-Gonzalo L, García-García D, Ajejas-Bazán MJ, Roquette-Viana MC, Tholl AD, et al. Attitudes and behaviors toward vaccination among nursing students from Spain and Portugal: a cross-sectional study. BMC Nurs. 2025;24:327. doi:10.1186/s12912-025-03627-3.

[39] Leshi E, Pagkozidis I, Exidari M, Gioula G, Chatzidimitriou M, Tirodimos I, et al. Mapping Adult Vaccine Confidence in Future Health Professionals: A Pilot Study among Undergraduate Students at Two Universities in Greece. Vaccines (Basel). 2024;12(7):742. doi:10.3390/vaccines12070742.

These additions provide up-to-date, directly relevant evidence on adult/life-course vaccination knowledge and attitudes among nursing and healthcare students, and incorporate both Spanish/local and international perspectives.

We believe these changes fully address your concern and significantly enhance the currency and rigor of the reference list.

We have also conducted a thorough verification of the entire manuscript and reference list to ensure full compliance with PLOS ONE’s documentation style. All in-text citations now match the bibliography exactly, with no omissions, duplications, or numbering discrepancies. Every reference has been formatted according to the journal’s requirements, including complete author lists (or “et al.” after six authors) and DOIs where available.

**Final Evaluation: Acceptance with Major Revisions**

**Reason:**

- **After reviewing the research, I believe it addresses a topic of clear scientific and applied importance and possesses a sound methodological foundation. However, several key aspects require development before its acceptance for publication. These include strengthening the theoretical framework and formulating the hypotheses more clearly, providing a more detailed presentation of the measurement tools and their psychometric properties, clarifying the rationale for the statistical analysis, and delving deeper into the discussion of the results and their connection to the literature in a more critical manner. Therefore, the study does not suffer from any definitive methodological shortcomings that would warrant rejection, but it requires substantial revisions to enhance its scientific quality and contribution. I thus recommend its acceptance conditional upon major revisions.**

We are grateful to the reviewer for the constructive evaluation and for recognizing the scientific and applied relevance of our study, as well as its solid methodological foundation. We confirm that all major aspects identified for improvement have been thoroughly addressed and revised in the current version of the manuscript, exactly as detailed in our point-by-point response:

- The theoretical framework has been strengthened and the three study hypotheses have been explicitly formulated and linked to the 5C model (Introduction).
- The measurement instrument (ad hoc questionnaire) is now presented with full detail on its development, adaptation process, pilot testing, and psychometric properties (Cronbach’s α, construct and discrimination validity) (Methods, subsection 2.2).
- The rationale for each statistical test has been clarified and justified in relation to the data distribution and study objectives (Methods, subsection 2.3).
- The Discussion has been substantially expanded with a more critical analysis of the results, their direct comparison with the most recent literature (including the newly added references), and clearer implications for nursing education and public health (Discussion, sections 4.1–4.5).

These targeted revisions have significantly enhanced the manuscript’s clarity, rigor, and contribution. We believe the paper now fully meets the journal’s standards and are confident it is ready for acceptance.

**Thank you very much**
