# Supplementary material for: Knowledge gaps and positive attitudes toward adult vaccination among nursing students: A Cross-sectional study
Source: PLoS One. 2026 May 26;21(5):e0350295. doi: 10.1371/journal.pone.0350295 (PMC13210314; doi:10.1371/journal.pone.0350295)
Supplement: Attachment — Submitted filename: Response to Reviewers.docx [file pone.0350295.s008.docx]

**Title:** Knowledge Gaps and Positive Attitudes Toward Adult Vaccination Among Nursing Students: A Cross-Sectional Study

PONE-D-26-02037

We wish to thank you greatly for the interest you have shown towards our study, for taking the time to review it and for your constructive comments.

All changes are indicated in blue in the manuscript and here.

Here follows a point-by-point response to your comments:

**Reviewer Comments:**

**Reviewer #2: The revised manuscript shows clear and substantial improvement. The authors have addressed the reviewers’ comments comprehensively, strengthening the theoretical framework, methodology, and discussion. The study design is appropriate, the statistical analysis is adequate for the research aims, and the conclusions are well supported by the data.

Data availability has been improved with the inclusion of supporting files, and ethical considerations are clearly reported. The manuscript is now well-structured, clearly written, and suitable for publication, with only minor editorial refinements if needed.**

We are grateful to the reviewer for the positive evaluation of our revised manuscript. We sincerely appreciate the reviewer’s recognition of the improvements made to the theoretical framework, methodology, discussion, and overall structure of the paper.

We are pleased that the reviewer finds the manuscript suitable for publication in PLOS ONE, with only minor editorial refinements if needed. We have carefully reviewed the manuscript once more to ensure clarity and adherence to journal style.

Thank you again for the supportive and encouraging comments, which have helped strengthen our work.

**Reviewer #3: Hello, dear authors.
MS ID: PONE-D-26-02037R1
Title: Knowledge Gaps and Positive Attitudes Toward Adult Vaccination Among Nursing Students: A Cross-Sectional Study in Alicante, Spain
Type: Research Article**

**Here are my recommendations regarding the mentioned manuscript:**

**Title:
• I suggest not mentioning the place in the title. Just “Knowledge Gaps and Positive Attitudes Toward Adult Vaccination Among Nursing Students: A Cross-Sectional Study”**

We thank the reviewer for this suggestion. We agree with the recommendation and have revised the title accordingly to:

“Knowledge Gaps and Positive Attitudes Toward Adult Vaccination Among Nursing Students: A Cross-Sectional Study”

**Abstract:
• In method subsection, mention statistical tests were used to inferential analysis, delete (Universidad de Alicante, CEU Cardenal Herrera, and Universidad Europea de Valencia), and mention statistical tests used.**

We thank the reviewer for this suggestion regarding the Abstract.

We have revised the Methods subsection of the Abstract as follows:

“Methods: An observational, descriptive, cross-sectional study was conducted among undergraduate nursing students in the province of Alicante, Spain, between April and May 2025. An ad hoc electronic survey with 19 closed-ended items was distributed to students from the three universities offering the Nursing degree in this province; 562 students participated. Differences across academic years were analyzed using Pearson’s χ² test for categorical variables and the Kruskal-Wallis test for the continuous variable age (non-normally distributed).”

We have removed the specific names of the three universities (“Universidad de Alicante, CEU Cardenal Herrera, and Universidad Europea de Valencia”) from the Abstract, as suggested, while retaining the information that the study was conducted in the province of Alicante with participation from the three institutions offering the Nursing degree. This maintains brevity and focus without losing essential context.

The full description of the statistical tests remains in the dedicated Statistical Analysis subsection of the main Methods section, in accordance with PLOS ONE guidelines.

These minor changes have been incorporated into the revised manuscript.

Thank you again for the constructive feedback, which has improved the clarity and conciseness of the Abstract.

**• In the results subsection, please clearly state your main findings in the inferential results with (p-value).**

We thank the reviewer for this suggestion.

We have revised the Results subsection of the Abstract to clearly highlight the main inferential findings with their corresponding p-values. The revised version now reads:

“Results: Overall, 39.1% of nursing students were unaware of the Spanish adult vaccination schedule, with significant differences across academic years (55.7% in first-year vs. 27.2% in fourth-year students; p < 0.001). Only 44.1% reported sufficient training on vaccines. Attitudes toward adult vaccination were predominantly positive: 61.4% highly favorable, 62.5% highly effective, and 66.7% supporting mandatory vaccination. Additionally, 65.5% would recommend all scheduled adult vaccines. However, 47.9% perceived resistance from other healthcare professionals when recommending vaccines (p < 0.001 for the increase across academic years).”

These changes emphasize the key statistically significant results while maintaining the abstract within the recommended word limit.

The updated Abstract has been incorporated into the revised manuscript.

Thank you again for the constructive feedback, which has improved the precision and clarity of the Abstract.

**Introduction:
• From line 111-115 no need to be mentioned in the introduction you can move it to methodology “tools”.
• No need to write hypothesis.**
We thank the reviewer for this suggestion.

The paragraph in question (previously lines 111–115) describes the 5C psychological antecedents of vaccine hesitancy model [25] and explains how this theoretical framework guided the selection of study variables, the design of the questionnaire items, and the formulation of the study hypotheses (H1–H3).

Following specific instructions from the journal during previous revision rounds, we placed the theoretical framework in the Introduction, as it provides the necessary rationale and foundation for the study objectives and hypotheses. Detailed information regarding the questionnaire development, adaptation from validated instruments, pilot study, validity, and reliability (the “tools”) is already correctly located in the Measures and Procedures subsection (Section 2.2) of the Methods.

We therefore respectfully maintain the current placement of the 5C model in the Introduction, as moving it would disrupt the logical flow of the manuscript and contradict previous editorial guidance from the journal.

We have not removed the hypotheses because they were included in the last revision at the express request of another reviewer.

Thank you again for the constructive feedback.

**Methodology:
• I prefer to divide methods to:
• Study design, setting, period, and sample size
• Study tool
• Study variables including code and scores
• Ethical approval and inform consent (summarize it).
• Statistical analysis.**

We thank the reviewer for this valuable suggestion to improve the organization and clarity of the Methods section.

We have restructured the Methods section according to the reviewer’s recommendation as follows:

**2.1. Study Design, Setting, Period, and Sample Size**

(Combines the original 2.1 with the sample size calculation and population description.)

**2.2. Study Tool (Questionnaire)**

(Includes the description of the ad hoc questionnaire, its adaptation from validated instruments, pilot study, validity, reliability, and structure.)

**2.3. Study Variables**

(New subsection: Describes the main variables, including sociodemographic, knowledge, attitudes/recommendation practices, and how they were coded/measured. Since the questionnaire does not generate composite numerical scores, we present the original response categories and coding.)

**2.4. Ethical Approval and Informed Consent**

(This section has not been summarized as it concerned requirements requested in previous reviews.)

**2.5. Statistical Analysis**

(Slightly revised for clarity, with the rationale for the chosen tests.)

The changes are visible in the revised manuscript with track changes.

Thank you again for this constructive recommendation, which has significantly improved the clarity of the manuscript.

**• Statistical analysis not well established. Why do you used Kruskal wails test?
• I suggest using regression test.**

**• I suggest to use parametric tests with presenting mean and standard deviation values.**

We thank the reviewer for these valuable statistical suggestions and for the opportunity to clarify our analytical approach.

The questionnaire used in this study (S1 File) consists primarily of categorical and ordinal variables (e.g., knowledge of the adult vaccination schedule [yes/no/do not know], level of training received [5-point ordinal scale], attitudes toward vaccine safety/efficacy [5-point ordinal scales], recommendation practices [categorical], and reasons for resistance [multiple-response categorical]). No composite numerical score for overall knowledge or attitudes could be derived, as the items assess distinct constructs rather than forming a continuous scale. Consequently, most variables are inherently categorical or ordinal, making contingency table analysis with Pearson’s χ² test the most appropriate method for examining associations across academic years, as reported in Tables 1–3.

The only continuous variable in the study is participant age. The Kolmogorov-Smirnov test indicated a non-normal distribution for age across academic years. Therefore, we correctly applied the non-parametric Kruskal-Wallis test to compare age distributions between the four academic years (Table 1), accompanied by presentation of means and standard deviations for descriptive purposes, which is standard practice even when using non-parametric tests.

We respectfully note that parametric tests (e.g., one-way ANOVA) would not be appropriate here due to the non-normal distribution of age and the categorical/ordinal nature of the remaining variables. Similarly, linear regression is not suitable, as the dependent variables of primary interest (knowledge items, attitudes, and recommendation practices) are categorical or ordinal rather than continuous. Logistic or ordinal regression could theoretically be considered for some binary/ordinal outcomes; however, given the descriptive and exploratory nature of the study (focused on describing differences across academic years in a specific regional population), the χ² tests for categorical variables and Kruskal-Wallis for age provide sufficient and transparent insight into the data without overcomplicating the analysis or risking overfitting in a sample of this size.

We have now explicitly clarified the rationale for the choice of statistical tests in the revised Statistical Analysis subsection as follows:

“Data are expressed as mean ± standard deviation for continuous variables (age) and as frequencies and percentages for categorical and ordinal variables. The normality of age was assessed using the Kolmogorov-Smirnov test. Differences in age across academic years were evaluated with the non-parametric Kruskal-Wallis test, given the non-normal distribution. For categorical and ordinal variables, associations across academic years were examined using contingency tables and Pearson’s χ² test. All analyses were performed using IBM SPSS Statistics version 29.0. Statistical significance was set at p < 0.05. There were no missing data, as all questionnaire items were mandatory.”

We believe this revision adequately addresses the reviewer’s concerns while maintaining methodological rigor and appropriateness for the data type. No additional regression analyses were performed, as they would not align with the study design or variable characteristics.

Thank you again for this constructive feedback, which has helped improve the clarity of the Methods section.

**• Not to categorize scores.**

We thank the reviewer for this comment regarding the categorization of scores.

The questionnaire employed in this study (see S1 File) was specifically designed with closed-ended categorical and ordinal items adapted from previously validated instruments. It does not generate a continuous or composite numerical score for the degree of knowledge (or attitudes) of each participant. The knowledge section consists of discrete items (e.g., awareness of the existence of the Spanish adult vaccination schedule [yes/no/do not know] and awareness of the specific vaccines included [yes/no]), which do not lend themselves to summation into a meaningful total score without introducing arbitrary weighting or assumptions that would compromise validity.

For this reason, we presented the results as frequencies and percentages according to the original response categories (Tables 2 and 3), which preserves the integrity of the data and allows transparent interpretation of each item. We did not create or impose artificial numerical scores or categorize derived continuous variables.

Nevertheless, we fully acknowledge the reviewer’s point and have now added the following statement as a limitation in the revised manuscript (Section 4.6, Limitations and Strengths of the Study):

“A further limitation is that the ad hoc questionnaire did not yield a continuous or composite score for knowledge or attitudes toward adult vaccination. Consequently, results are presented as categorical frequencies and percentages rather than summarized numerical scores. This approach reflects the structure of the instrument but limits the possibility of more nuanced quantitative analyses (e.g., correlation or regression with total scores). Future studies should consider validated instruments that provide continuous knowledge and attitude scores when such metrics are required.”

We believe this addition adequately addresses the reviewer’s concern by transparently recognizing the constraint while justifying the analytical decisions made.

Thank you again for this constructive feedback, which has strengthened the methodological transparency of the manuscript.

**Results:
• After conducting tests, which I suggested, results may change for the better.
• I suggest to create a bar chart presenting participants level of knowledge.**

We thank the reviewer for this suggestion.

As detailed in our previous response and in the revised Statistical Analysis section, the questionnaire used in this study (S1 File) generates only categorical and ordinal variables and does not produce a continuous or composite numerical score that quantifies the overall “level of knowledge” of each participant. Therefore, it is not possible to perform the regression analyses or parametric tests previously suggested by the reviewer, nor to create a bar chart that meaningfully represents a quantified “level of knowledge,” as no such aggregated score exists.

We have already addressed this limitation explicitly in the revised manuscript (Section 4.6, Limitations and Strengths of the Study), where we state that the absence of a continuous knowledge score restricts more advanced quantitative analyses and that results are appropriately presented as frequencies and percentages for each individual item (Tables 2 and 3).

We believe the current presentation of results remains the most accurate and transparent approach given the nature of the data collected.

Thank you once again for the constructive comments.

**Discussion:
• No need to introduction paragraph for discussion.**

**• Do not re-write the results in the discussion.**

**• Do not divide discussion to subsections.**

**• Just discuss your main results with previous study results with providing justifications for the observed similarities and differences in the findings.**

We thank the reviewer for their suggestions.

We have removed the first paragraph. The titles of the subsections have also been removed.

We have carefully revised the entire Discussion section and removed all specific numerical data from our own study that were previously restated from the Results section. The Discussion now focuses on the interpretation, comparison with the literature, and implications of the findings rather than repeating the results.

**Conclusion:
• Looks good.**

Thank you

**References:
• Looks good.**

Thank you
